# Aspiration Is Associated with Poor Treatment Response in Pediatric Pulmonary Vein Stenosis

**DOI:** 10.3390/children8090783

**Published:** 2021-09-07

**Authors:** Maria Niccum, Ryan Callahan, Kimberlee Gauvreau, Kathy J. Jenkins

**Affiliations:** 1 Department of Pediatrics, Boston Children’s Hospital and Harvard Medical School, 300 Longwood Avenue, Boston, MA 02115, USA; niccumm@chop.edu; 2 Department of Cardiology, Boston Children’s Hospital and Harvard Medical School, 300 Longwood Avenue, Boston, MA 02115, USA; Ryan.Callahan@cardio.chboston.org (R.C.); Kimberlee.Gauvreau@cardio.chboston.org (K.G.)

**Keywords:** congenital heart disease, aspiration, outcome

## Abstract

Intraluminal pulmonary vein stenosis is a disease with significant morbidity and mortality, though recent progress has been made using multimodal therapy with antiproliferative agents. The aim of this study was to evaluate the association between aspiration and poor treatment response in patients with intraluminal pulmonary vein stenosis. A retrospective, single-center cohort analysis was performed of patients treated with a combination of imatinib mesylate and multimodal anatomic relief between March 2009 and November 2019. Analysis focused on 2-ventricle patients due to small numbers and clinical heterogeneity of single ventricle patients. Among the 84 patients included, 15 had single ventricle physiology and 69 had 2-ventricle physiology. Among the 2-ventricle group, multivariable analysis revealed that patients with clinical aspiration had nearly five times higher odds of poor treatment response than patients without aspiration (OR 4.85, 95% CI [1.37, 17.2], *p* = 0.014). Furthermore, male patients had higher odds of poor treatment response than their female counterparts (OR 3.67, 95% CI [1.04, 12.9], *p* = 0.043). Aspiration is a novel, potentially modifiable risk factor for poor treatment response in pediatric multi-vessel intraluminal pulmonary vein stenosis in patients with 2-ventricle physiology.

## 1. Introduction

Intraluminal pulmonary vein stenosis (PVS) is a rare disease characterized by recurrent pulmonary vein obstruction due to overactivity of myofibroblast-like cells within the pulmonary vein wall [1,2]. PVS can lead to hypoxemia, pulmonary arterial hypertension, right ventricular failure, and death. Recent advances in surgical [3,4,5] and catheterization techniques [6,7,8] and use of targeted agents to suppress cellular activity [9,10,11] have markedly improved survival. Causal factors definitively leading to cellular overactivity remain unknown. 

Even with aggressive, multimodal therapy, response to treatment varies among patients [6,12,13,14,15]. While some patients experience disease stabilization over time (evidenced by less frequent need for pulmonary vein re-interventions), a substantial proportion of patients continue to require frequent re-interventions despite maximal therapy. Several studies have evaluated risk factors for poor outcomes in PVS and have shown that patients with bilateral pulmonary vein involvement [13] and those who are smaller and younger at diagnosis have a higher risk of mortality [3]. Additionally, patients with lung disease have lower rates of disease stabilization, defined as no re-interventions at six months, when compared with patients without lung disease [9]. Differences in risk for various types of lung disease have not been characterized. However, since some types of lung disease are treatable, a better understanding of the role of lung disease may yield an opportune target for interventions aiming to improve PVS outcomes. To our knowledge, the relationship between aspiration associated lung disease and PVS outcomes has not been studied.

We have observed anecdotally that patients with PVS who show signs of oropharyngeal aspiration have worse outcomes, as evidenced by increased need for re-interventions and lower rates of disease stabilization. Untreated, chronic aspiration can cause significant lung damage, both by direct injury to lung parenchyma, and by repeated episodes of aspiration pneumonia [16]. We hypothesized that oropharyngeal aspiration may contribute to recurrence of PVS via damage to lung parenchyma and associated changes in pulmonary vein mechanics. Our aim for this study was to characterize the relationship between aspiration and PVS treatment response in order to identify a potential modifiable risk factor that could improve PVS outcomes.

## 2. Materials and Methods

### 2.1. Study Design

We performed a single center retrospective cohort analysis of all patients with multi-vessel PVS (gradient ≥ 4 mmHg by echocardiography or catheterization) and disease recurrence who received imatinib mesylate (Gleevec®, Novartis Inc., Basel, Switzerland) drug therapy from March 2009 to November 2019 as a part of a multimodal treatment plan. Patients who received bevacizumab (Avastin®, Genentech Inc., South San Francisco, CA, USA) in addition to imatinib mesylate at the start of therapy were also included. Patients were identified using our institutional PVS database and included those who participated in our prospective study [9] and those treated after the trial stopped enrollment. There have been no substantive changes to our therapeutic protocol for patients with PVS since the conclusion of the trial. Patients were categorized as either having single ventricle or 2-ventricle physiology at start of imatinib mesylate therapy. Patients who underwent a bi-ventricular conversion procedure prior to initiation of therapy were included in the 2-ventricle group. Only patients who had longitudinal follow-up at Boston Children’s Hospital were included. This study was approved by the Boston Children’s Hospital Institutional Review Board and was performed in accordance with the Declaration of Helsinki.

### 2.2. Outcomes and Data Collection

The primary outcome for this study was therapeutic response, assessed 12 months after initiation of imatinib mesylate, similar to the 48-week primary endpoint in the prospective clinical trial. For the current study, poor response to therapy was defined by any of the following: ongoing requirement of surgical and/or catheter re-interventions for pulmonary vein restenosis at a frequency of ≤12 weeks, PVS-related mortality or listing for lung transplantation, or the need to add bevacizumab to the treatment regimen due to PVS progression (≥2 veins with: new obstruction in a previously unaffected vein, proximal disease extending distally >5 mm, or new vessel atresia) [9]. Patients not meeting any of the above criteria were categorized as having good response to therapy. Data were collected retrospectively from review of the institutional PVS registry and patient medical records.

Clinically significant aspiration was defined as having any of the following at the time of initiation of imatinib: clinical aspiration observed by the speech and language pathology (SLP) consultation team, documented aspiration on videofluoroscopic swallow study (VFSS), or use of post-pyloric feeds due to intractable vomiting/feeding intolerance. Clinically significant aspiration had not been studied as a part of the clinical trial and was assessed for this study after careful review of SLP consultation notes, VFSS reports, and daily physician progress notes. Aspiration risk was subsequently managed at the discretion of the treatment team. Potential confounders included sex, prematurity (gestational age <37 weeks), presence of a genetic syndrome, presence and type of congenital heart disease, anatomy of pulmonary venous connections, bilateral disease at diagnosis, presence of lung disease other than aspiration (bronchopulmonary dysplasia, lung hypoplasia, airway malacia, interstitial lung disease, and/or congenital lobar emphysema), age at start of imatinib mesylate, and pulmonary artery to aorta (PA/Ao) systolic pressure ratio at the start of imatinib mesylate. 

### 2.3. Statistical Analysis

Patient characteristics and outcomes were summarized using frequencies and percentages for categorical variables, and medians with interquartile ranges for continuous variables. Analysis of the effect of aspiration on poor treatment response was restricted to patients with 2-ventricle physiology because the single ventricle group was small, heterogeneous, and clinical trajectory may have been different (including catheterization and reintervention frequency, for example) due to disease complexity. 

Logistic regression was used to quantify the relationship between poor treatment response and clinically significant aspiration, first unadjusted, and then in a multivariable model adjusting for known confounders and risk factors found to be significantly associated with the outcome. Odds ratios were estimated with 95% confidence intervals.

Among the 2-ventricle group, sub-analyses were performed comparing differences in individual components of the composite outcome variable between patients with and without aspiration. Sub-analyses were also performed comparing the differences in individual aspiration variables between the poor and good treatment response groups. Comparisons were performed using Fisher’s exact test and the Wilcoxon rank sum test. Analyses were performed using Stata version 16 (StataCorp LLC, College Station, TX, USA).

## 3. Results

### 3.1. Patient Population

We identified 104 patients who received imatinib mesylate for multivessel PVS, of whom 84 had longitudinal follow-up and comprised the study population. Of these 84 patients, 15 had single ventricle physiology at the start of imatinib mesylate therapy, and 69 had 2-ventricle physiology. For the 2-ventricle patients, the median age at start of imatinib mesylate therapy was 0.6 years (0.2, 4.3). Bilateral disease was present at diagnosis in 86% (59/69), and the median PA/Ao systolic ratio at start of treatment was 0.8 (0.4, 2.1) (Table 1). 

### 3.2. Demographics and Outcomes for 2-Ventricle Patients

Of the 69 patients with 2-ventricle physiology, 19 (28%) met criteria for poor response to therapy. Patients who had poor response to therapy were significantly more likely to have clinically significant aspiration (74%, 14/19 vs. 34%, 17/50, *p* = 0.006). Patients with poor response were also more likely to be male (74%, 14/19 vs. 40%, 20/50, *p* = 0.016). No significant differences were found between groups with respect to prematurity, genetic diagnosis, presence and type of CHD, anatomy of pulmonary venous connections, presence of bilateral disease at diagnosis, lung disease, PA/Ao systolic ratio at start of treatment, and age at start of treatment with imatinib mesylate (Table 1). 

### 3.3. Multivariate Risk Factors for Poor Treatment Response

In univariate logistic regression, patients with aspiration had an odds of poor treatment response that was 5.44 times higher than patients without aspiration (95% CI [1.68, 17.6], *p* = 0.005) (Table 2). After adjusting for confounders, aspiration remained significantly associated with poor treatment response, with an odds ratio of 4.85 (95% CI [1.37, 17.2], *p* = 0.014). Additionally, males had an odds of poor treatment response that was 3.67 times higher than females (95% CI [1.04, 12.9], *p* = 0.043) (Table 3). 

### 3.4. Individual Outcomes in Patients with and without Aspiration

To further assess the impact of aspiration on specific treatment outcomes, additional analyses were performed and are detailed in Table 4. Patients with aspiration were significantly more likely to have poor response to treatment (45%, 14/31 vs. 13%, 5/38, *p* = 0.006), and were more likely to have died before completing 12 months of imatinib mesylate therapy (19%, 6/31 vs. 3%, 1/38, *p* = 0.04). Patients with aspiration were more likely to require pulmonary vein re-interventions at a frequency of ≤12 weeks after 12 months of therapy (13%, 4/31 vs. 5%, 3/38, *p* = 0.012). Patients with aspiration were also more likely to have received bevacizumab therapy either at the start of treatment or added to the treatment regime due to disease progression (*p* = 0.009). There was no significant difference in the number of patients who were listed for lung transplant (23%, 7/31 vs. 5%, 2/38, *p* = 0.068) or underwent lung transplantation (13%, 4/31 vs. 3%, 1/38, *p* = 0.17) in the first year of therapy. Lastly, patients with aspiration were more likely to have non-aspiration related lung disease (77%, 24/31 vs. 53%, 20/38, *p* = 0.045).

### 3.5. Individual Components of Aspiration

Individual variables included in the definition of “clinically significant aspiration” were evaluated in Table 5. Patients with poor response to treatment were significantly more likely to have aspiration observed on bedside SLP evaluation (47%, 9/19 vs. 18%, 9/50, *p* = 0.029), aspiration observed on VFSS (42%, 8/19 vs. 16%, 8/50, *p* = 0.03), and significant feeding intolerance requiring post-pyloric feeds (58%, 11/19 vs. 18%, 9/50, *p* = 0.002). 

### 3.6. Sex Differences

Additional analysis was performed comparing male and female patients to explore differences between these groups (Supplemental Table S1). Males were more likely to require re-intervention at a frequency of ≤12 weeks after 12 months of imatinib mesylate therapy (18%, 6/34 vs. 0%, 0/35, *p* = 0.004). No sex differences were found for any other clinical variables.

## 4. Discussion

### 4.1. Aspiration and PVS Outcomes

Despite recent advances in both pharmacologic and anatomic management strategies, PVS remains a disease with significant morbidity and mortality [6,9,12,13,14,15,17]. Identification of modifiable risk factors for PVS recurrence and PVS treatment failure is an area of great need. By recognizing patients at risk of poor treatment response early in the disease course, strategies can be implemented to reduce risk and improve overall outcomes.

In our study, we identified a strong association between pretreatment clinical aspiration and poor response to multi-modal therapy in pediatric patients with recurrent multi-vessel intraluminal PVS. This finding was both highly significant and of large magnitude. Patients who aspirate were *nearly five times* more likely to have poor treatment response when compared with patients without aspiration, even when controlling for factors known to impact PVS severity such as bilateral disease, age, and the presence of lung disease. Furthermore, patients with aspiration were significantly more likely to die in the first year of therapy. While patients received treatment for aspiration at the discretion of treating providers, our findings suggest that clinically significant aspiration is a potentially important modifiable clinical risk factor associated with PVS outcome. Although patients with gastroesophageal reflux or oromotor dyscoordination commonly receive medications or thickened feeds, symptom thresholds for more aggressive strategies such as airway evaluation, discontinuation of oral feedings, institution of jejunal feeding, or Nissen fundoplication vary among providers. Furthermore, aspiration is a dynamic process, and frequent re-assessments are necessary to determine whether an intervention previously implemented remains successful at later time points. In light of the possible association between aspiration and poor response to PVS therapy, more aggressive strategies could be considered to mitigate aspiration in this population.

### 4.2. Proposed Mechanism

Chronic oropharyngeal aspiration is associated with significant pulmonary disease [16,18,19,20,21]. Autopsy specimens taken from patients with chronic aspiration predominantly show bronchiolar fibroblast proliferation and granulation tissue formation, inflammatory bronchiolitis, pneumonia, and occasionally interstitial pulmonary fibrosis [21]. In mouse models of aspiration, these histopathologic findings have been associated with decreased lung elastance, increased airway resistance, de-recruitment of lung tissue, and airway hyperresponsiveness [19,22,23,24]. In a study of infants with swallowing dysfunction, both obstructive and restrictive patterns of lung injury were seen on pulmonary function testing [25], and others have documented bronchoconstriction, airway hyperreactivity, and hyperinflation in children with chronic pulmonary aspiration [18,26]. 

**Figure 1:**
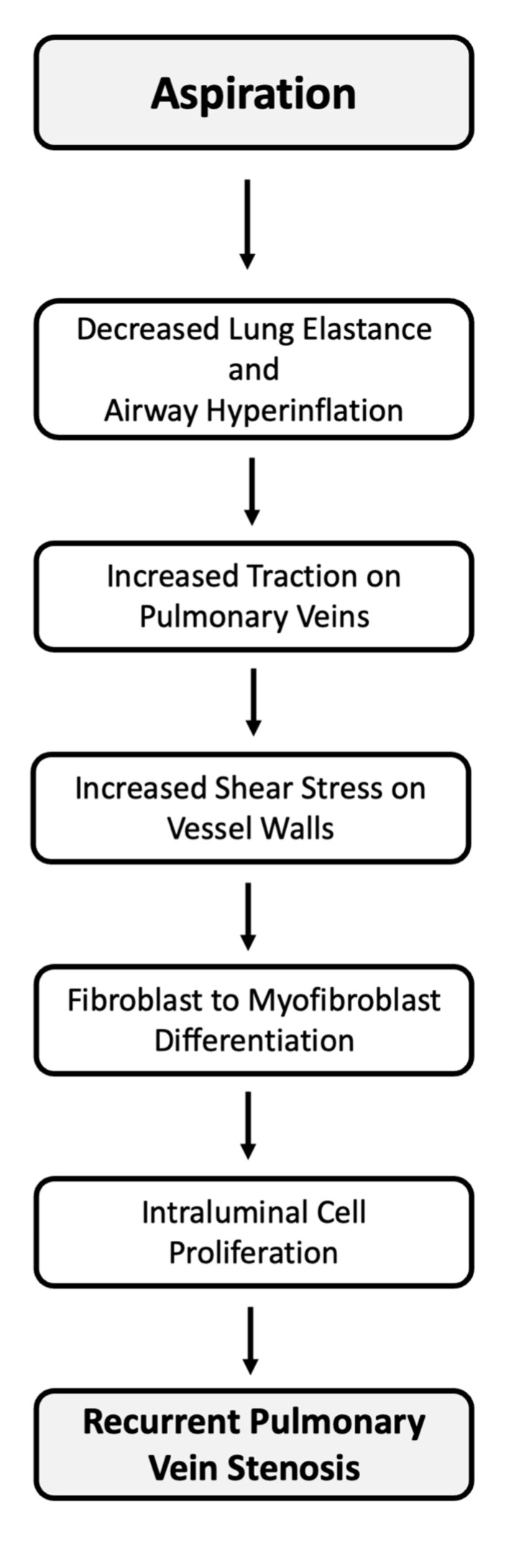
Proposed Mechanism of PVS Recurrence. Schematic outlining a potential mechanism by which aspiration and male sex lead to PVS recurrence.

We speculate that mechanical changes associated with chronic aspiration and hyperinflation may directly impact the cells that comprise pulmonary veins, leading to hyperproliferation and intraluminal obliteration (Figure 1). Lung hyperinflation and increased airway resistance resulting from chronic aspiration may generate increased traction on the pulmonary veins, sending mechanical signals to vessel fibroblasts. Increased vascular wall shear stress has previously been implicated in pulmonary vein stenosis [27]. Mechanical traction in the extracellular matrix can stimulate fibroblast to myofibroblast differentiation and promote myofibroblast proliferation through a TGF-β1-dependent mechanism [28,29]. Our group has previously implicated myofibroblast-like cell proliferation in PVS [2,30], and our current treatment is aimed at tyrosine-kinase receptor inhibition using targeted anti-proliferative pharmacotherapy [9]. If unfavorable mechanical forces generated by aspiration lung disease lead to a strong pro-proliferation signal, this could explain our finding that patients with clinically significant aspiration are at higher risk of treatment failure, and perhaps suggest a causal role for aspiration in PVS. Notably, although lung disease was not associated with vein restenosis in a sub-analysis [31] of our clinical trial population, the definition of lung disease used in the clinical trial focused on chronic lung disease, not aspiration, and was much broader, including chronic lung disease of prematurity, airway malacia, lung hypoplasia, interstitial lung disease, congenital lobar emphysema, and aspiration lung disease. Thus, the effect of aspiration lung disease may have been diluted. In contrast, this study focused exclusively on aspiration, and included a much larger population of patients with PVS treated with imatinib mesylate, thereby increasing the likelihood of detecting a significant association.

### 4.3. Male Sex and PVS Outcomes

One unexpected finding in our analysis was the association of male sex with poor treatment response. This association remained significant in our multivariable analysis, indicating an effect of sex that is independent of aspiration. Furthermore, the effect size was large; male patients were 3.7 times more likely to respond poorly to treatment than females. Sex differences in various respiratory outcomes have been described previously in the pediatric population. A robust body of evidence has shown that both premature and term male infants are at higher risk of respiratory distress syndrome and respiratory infections, and are more likely to require mechanical ventilation and supplemental oxygen after birth [32,33,34,35,36]. Interestingly, in a population of formerly premature children evaluated at one year of age, males were more likely to have higher functional residual capacity, airway resistance, hyperinflation, and evidence of air trapping than their female counterparts [36]. Taken together, these findings could suggest a similar pathophysiologic mechanism by which male sex negatively impacts treatment response in patients with PVS. However, our study was not designed to evaluate this risk factor, and more targeted studies are needed to further explore this relationship.

### 4.4. Strengths and Limitations

This study has several strengths. First, our definition of aspiration was very strict and included only patients with significant symptoms. Similarly, our definition of poor treatment response was limited to patients with objective findings such as death, frequent reinterventions, or lung transplantation at 12 months. Additionally, the cohort study design enabled us to target the entire population of patients treated with contemporary multimodal therapy including imatinib mesylate at our institution, which allowed us to calculate the true effect size of aspiration on treatment responsiveness. Our study also has several limitations. Due to the retrospective study design, there were limitations in how aspiration was identified and classified, which required us to only include more severe cases. Furthermore, treatment for aspiration was not standardized, and our study was not designed to evaluate optimum strategies for management. Last, our center is a quaternary care referral center and many of our patients with PVS were cared for at other centers prior to or after receiving care at our institution. This study only includes patients who were followed longitudinally at our institution, and thus may be biased towards the most severe cases and may not be representative of all patients with PVS.

## 5. Conclusions

In summary, two novel risk factors for poor response to multimodal PVS treatment including imatinib mesylate as an antiproliferative agent were identified for patients with 2-ventricle physiology: clinical aspiration and male sex. Despite limitations in our retrospective design, these findings have identified an important role for clinical aspiration in PVS. The effect of aspiration and male sex on PVS outcomes both warrant further study. Specifically, future studies are needed to determine whether early identification and more aggressive treatment of aspiration can improve PVS outcomes. Future studies are also needed to evaluate the role of aspiration in higher risk patients with single ventricle. In the meantime, assessment for aspiration and aggressive clinical treatment should be considered for all patients with significant PVS. 

## 6. Clinical Outlook

- 2-ventricle patients with PVS and aspiration may respond poorly to multimodal PVS therapy
- Aggressive aspiration surveillance and management is warranted in 2-ventricle patients with PVS
- Close follow-up of 2-ventricle patients with PVS and aspiration may lead to earlier identification of poor responders

## Figures and Tables

**Table 1:** Demographics for 2-Ventricle Patients.

	Poor Treatment Response (*n* = 19)	Good Treatment Response (*n* = 50)	*p* Value
Male Sex	14 (74%)	20 (40%)	0.016 *
Premature	11 (58%)	21 (43%)	0.29
Genetic Syndrome	4 (21%)	14 (28%)	0.76
Presence of CHD			0.40
None	4 (21%)	4 (8%)	
ASD/VSD/AVSD/PDA	8 (42%)	18 (36%)	
TAPVC/PAPVC	3 (16%)	11 (22%)	
All others	4 (21%)	17 (34%)	
Pulmonary Venous Connections			0.28
Normal	15 (79%)	32 (64%)	
PAPVC	0 (0%)	7 (14%)	
TAPVC	4 (21%)	11 (22%)	
Bilateral Disease at Diagnosis	17 (89%)	42 (84%)	0.72
Lung Disease	15 (79%)	29 (58%)	0.16
Age at Start of Treatment (years)	0.8 (0.2, 2.0)	0.6 (0.2, 4.3)	0.97
PA/Ao Systolic Ratio at Start of Treatment	1.0 (0.4, 2.0)	0.7 (0.4, 2.1)	0.059
Clinically Significant Aspiration	14 (74%)	17 (34%)	0.006 *

Values shown are either number (percent) or median (interquartile range). Abbreviations: CHD, congenital heart disease; ASD, atrial septal defect; VSD, ventricular septal defect; AVSD, atrioventricular septal defect; PDA, patent ductus arteriosus; TAPVC, total anomalous pulmonary venous connection; PAPVC, partial anomalous pulmonary venous connection; PA, pulmonary artery; Ao, aorta. * *p* < 0.05.

**Table 2:** Unadjusted Model.

	Odds Ratio	95% Confidence Interval	*p* Value
Clinically Significant Aspiration	5.44	(1.68, 17.6)	0.005 *

* *p* < 0.05.

**Table 3:** Multivariable Model.

	Odds Ratio	95% Confidence Interval	*p* Value
Clinically Significant Aspiration	4.85	(1.37, 17.2)	0.014 *
Age at Start of Treatment (years)	1.39	(0.50, 3.92)	0.53
Male Sex	3.67	(1.04, 12.9)	0.043 *
Bilateral Disease at Diagnosis	1.48	(0.23, 9.50)	0.68
Lung Disease	1.84	(0.47, 7.26)	0.38

* *p* < 0.05.

**Table 4:** Individual Treatment Outcomes for 2-Ventricle Patients.

	Patients Who Aspirate (*n* = 31)	Patients Who Do Not Aspirate (*n* = 38)	*p* Value
Lung Disease	24 (77%)	20 (53%)	0.045 *
Frequency of Interventions after 12 mo of Imatinib Mesylate			0.012 *
≤12 weeks	4 (13%)	2 (5%)	
>12 weeks	20 (65%)	35 (92%)	
Not reported	7 (23%)	1 (3%)	
Use of Bevacizumab			0.009 *
Received at Initial Treatment	7 (23%)	3 (8%)	
Added during first 12 mo of Imatinib	6 (19%)	1 (3%)	
None	18 (58%)	34 (89%)	
Listed for Lung Transplant during first 12 mo of Imatinib Mesylate	7 (23%)	2 (5%)	0.068
Underwent Lung Transplant during first 12 mo of Imatinib Mesylate	4 (13%)	1 (3%)	0.17
Alive after 12 mo of Imatinib Mesylate	25 (81%)	37 (97%)	0.040 *
Poor Response to Therapy	14 (45%)	5 (13%)	0.006 *

Values shown are number (percent). * *p* < 0.05.

**Table 5:** Individual Aspiration Variables for 2-Ventricle Patients.

	Poor Treatment Response (*n* = 19)	Good Treatment Response (*n* = 50)	*p* Value
Aspiration by SLP Evaluation	9 (47%)	9 (18%)	0.029 *
Aspiration by VFSS	8 (42%)	8 (16%)	0.030 *
Intractable Feeding Intolerance Requiring Post-Pyloric Feeds	11 (58%)	9 (18%)	0.002 *

Values shown are number (percent). Abbreviations: SLP, speech language pathology; VFSS, videofluoroscopic swallow study. * *p* < 0.05.

## Data Availability

The data presented in this study are available on request from the corresponding author. The data are not publicly available in order to maintain patient privacy.

## Supplementary Materials

The following are available online at https://www.mdpi.com/article/10.3390/children8090783/s1, Table S1: Sex Differences.
