# Supplementary material for: Aspiration Is Associated with Poor Treatment Response in Pediatric Pulmonary Vein Stenosis
Source: Children (Basel). 2021 Sep 7;8(9):783. doi: 10.3390/children8090783 (PMC8471903; doi:10.3390/children8090783)
Supplement: Supplementary file 1 [file children-08-00783-s001.zip › children-1363256-supplementary.pdf]

Supplemental Table S1. Sex Differences.

	**Males (*n* = 34)**	**Females (*n* = 35)**	***p* value**
Premature	15 (45%)	17 (49%)	0.81
Genetic Syndrome	6 (18%)	12 (34%)	0.17
Presence of CHD			
None	4 (12%)	4 (11%)	0.66
ASD/VSD/AVSD/PDA	12 (35%)	14 (40%)	
TAPVC/PAPVC	9 (26%)	5 (14%)	
All others	9 (26%)	12 (34%)	
Pulmonary Venous Connections			
Normal	22 (65%)	25 (71%)	0.70
PAPVC	3 (9%)	4 (11%)	
TAPVC	9 (26%)	6 (17%)	
Bilateral Disease at Diagnosis	31 (91%)	28 (80%)	0.31
Lung Disease	24 (71%)	20 (57%)	0.32
Age at Start of Treatment (years)	0.6 (0.4, 1.0)	0.6 (0.4, 1.1)	0.89
PA/Ao Systolic Ratio at Start of Treatment	0.8 (0.6, 1.0)	0.7 (0.5, 1.0)	0.80
Clinically Significant Aspiration	18 (53%)	13 (37%)	0.23
Frequency of Interventions after 12 mo of Imatinib Mesylate			0.004*
≤12 weeks	6 (18%)	0 (0%)	
>12 weeks	22 (65%)	33 (94%)	
Not reported	6 (18%)	2 (6%)	
Use of Bevacizumab			0.31
Received at Initial Treatment	6 (18%)	4 (11%)	
Added during first 12 mo of Imatinib Mesylate	5 (15%)	2 (6%)	
None	23 (68%)	29 (83%)	
Listed for Lung Transplant during first 12 mo of Imatinib Mesylate	5 (15%)	4 (11%)	0.73
Underwent Lung Transplant during first 12 mo of Imatinib Mesylate	2 (6%)	3 (9%)	1.0
Alive after 12 mo on Imatinib Mesylate	29 (85%)	33 (94%)	0.26
Poor Treatment Response	14 (41%)	5 (14%)	0.016*

Values shown are either number (percent) or median (interquartile range). Abbreviations: CHD, congenital heart disease; ASD, atrial septal defect; VSD, ventricular septal defect; AVSD, atrioventricular septal defect; PDA, patent ductus arteriosus; TAPVC, total anomalous pulmonary venous connection; PAPVC, partial anomalous pulmonary venous connection; PA, pulmonary artery; Ao, aorta.
